# Broccoli-Derived Peptides and Leucine in Combination Ameliorate D-Galactose-Induced Sarcopenia in Mice

**DOI:** 10.3390/nu18121997

**Published:** 2026-06-19

**Authors:** Kexin Yuan, Wenbin Wu, Ning Su, Mingyang Cui, Jingyi Qi, Yang Zhang, Zhengyang Zhang, Peng An, Junjie Luo, Yongting Luo

**Affiliations:** Department of Nutrition and Health, China Agricultural University, Beijing 100193, China; yuankexin0406@sina.com (K.Y.); wwb091828@163.com (W.W.);

**Keywords:** sarcopenia, broccoli-derived peptides, leucine, muscle mass, muscle strength, protein metabolism

## Abstract

Background: Sarcopenia is an age-related disorder characterized by loss of muscle mass, strength, and function, driven by oxidative stress, chronic inflammation, and protein imbalance. Broccoli-derived peptides (BDP) exert anti-inflammatory and myofiber-protective effects, while leucine regulates energy metabolism and redox balance. Methods: We established a D-galactose aging mouse model and treated mice with BDP alone, leucine alone, or their combination for 8 weeks. Lean mass, muscle index, grip strength, endurance, and treadmill capacity were detected, and atrophic, disorganized myofibers were observed through histology. RNA-seq was applied to screen differential signaling pathways, and qPCR was used to verify related gene expression levels. Results: D-galactose caused marked deficits in lean mass, muscle index, grip strength, endurance, and treadmill capacity, accompanied by atrophic and disorganized myofibers. Single BDP or leucine partially reversed these deficits, but the combination produced the most robust improvements. RNA-seq revealed that BDP enriched actin, chemokine, and TNF pathways; leucine enriched Apelin and ECM pathways; while the combination uniquely regulated MAPK signaling. qPCR confirmed that co-administration optimally upregulated myogenic drivers (*Myod1*, *Myog*, *Mef2c*), suppressed catabolic/inflammatory mediators (*Mstn*, *Tnf*, *Cxcl10*), and restored metabolic/adhesive regulators (*Sirt3*, *Aplnr*, *Icam1*). Conclusions: BDP and leucine show superior efficacy in ameliorating sarcopenia, through multimodal regulation of multiple signaling pathways, offering a promising plant-based nutritional strategy against age-related muscle decline.

## 1. Introduction

Sarcopenia is an age-related progressive disorder characterized by the accelerated loss of skeletal muscle mass, strength, and function, which significantly contributes to physical disability, frailty, falls, fractures, and increased mortality in the elderly population [1]. With the global population aging rapidly, the burden of sarcopenia on healthcare systems continues to escalate [2]. Epidemiological studies indicate that sarcopenia affects approximately 5–10% of the general population, with prevalence rates rising to 10–27% in adults aged over 60 years, and this prevalence is expected to increase markedly in the coming decades [1,3]. This makes the development of effective preventive and therapeutic strategies an urgent public health priority [4]. Data from specific populations, such as Japanese older adults, have further confirmed the high prevalence and adverse outcomes of sarcopenia [4].

The pathogenesis of sarcopenia is multifactorial, involving oxidative stress, mitochondrial dysfunction, chronic low-grade inflammation (“inflammaging”), an imbalance between protein synthesis and degradation, and impaired satellite cell regenerative capacity [5]. Among these mechanisms, oxidative stress is considered a core driver of sarcopenia onset and progression [6]. During aging, excessive accumulation of reactive oxygen species (ROS) from the mitochondrial respiratory chain in skeletal muscle overwhelms the endogenous antioxidant defense system, leading to lipid peroxidation, protein carbonylation, DNA damage, and activation of the ubiquitin–proteasome pathway and apoptotic signaling, ultimately resulting in myofiber atrophy and loss [7,8]. Moreover, ROS can activate pro-inflammatory pathways such as NF-κB and promote the release of pro-inflammatory cytokines, further exacerbating muscle catabolism [6]. Therefore, alleviating oxidative stress and chronic inflammation through exogenous antioxidants and anti-inflammatory agents has emerged as an important strategy for sarcopenia prevention and treatment [5]. A comprehensive review has also highlighted the central role of oxidative stress in human pathology and aging [9].

The D-galactose-induced aging mouse model has been widely used to investigate sarcopenia, as it recapitulates key features of age-related muscle atrophy including oxidative stress, mitochondrial dysfunction, and protein homeostasis imbalance [10]. It should be noted that the D-galactose model is an accelerated aging model primarily driven by oxidative stress and mitochondrial dysfunction, rather than a full recapitulation of naturally occurring geriatric sarcopenia. Nevertheless, this model reproduces key sarcopenic features including muscle mass loss, decreased grip strength, and myofiber atrophy, making it a widely accepted and practical tool for initial efficacy screening of nutritional interventions against age-related muscle decline. Rodent models have proven valuable for evaluating the effects of nutritional and non-nutritional interventions on skeletal muscle health during aging [11]. Studies using this model have demonstrated that various nutritional interventions can significantly improve muscle mass, exercise endurance, and grip strength in aging mice [10,11]. Moreover, compared with intact proteins, peptides have been shown to possess higher absorption and utilization rates, which may make them a highly attractive nutritional intervention strategy for sarcopenia [12,13].

Leucine, one of the three branched-chain amino acids, is the most critical nutritional signaling molecule regulating skeletal muscle protein metabolism [14]. Unlike other amino acids, leucine directly activates the mechanistic target of rapamycin complex 1 (mTORC1) signaling pathway, initiating muscle protein translation and synthesis by phosphorylating downstream effectors [15,16]. Elderly patients with sarcopenia often exhibit “anabolic resistance”—a blunted muscle protein synthetic response to amino acid and protein intake [17]. Leucine supplementation can effectively overcome this resistance and enhance muscle protein synthesis [14]. Clinical evidence supports this notion: a randomized controlled trial in elderly patients with chronic kidney disease showed that daily supplementation with leucine-enriched branched-chain amino acids for 12 weeks significantly increased lean muscle mass [18]. Furthermore, a recent meta-analysis of ten randomized controlled trials found that older sarcopenic patients receiving leucine supplementation showed significant improvements in handgrip strength, appendicular skeletal muscle mass index, and gait speed [19]. However, the capacity of leucine to exert beneficial therapeutic effects in older sarcopenic individuals may be limited when used alone without concurrent additional therapy [20].

Broccoli (*Brassica oleracea* var. *italica*) is among the cruciferous vegetables richest in bioactive components, and its consumption has been associated with antioxidant, anticancer, antimicrobial, and anti-inflammatory activities [21]. Plant-derived bioactive peptides exhibit anti-inflammatory, antioxidant, and immunomodulatory properties, making them promising candidates for therapeutic applications [22,23]. Notably, antimicrobial peptides derived from broccoli have been shown to target oxidative stress pathways and inflammatory mediators, suggesting that the anti-inflammatory and antioxidant activities of broccoli-derived compounds may be relevant for muscle health [21]. More importantly, recent studies have demonstrated that plant-based bioactive compounds can prevent sarcopenia through multiple mechanisms, including anti-atrophy effects, oxidative damage prevention, enhanced myogenesis, and anti-inflammatory activity [24,25]. In particular, a comprehensive review on food-derived anti-sarcopenic peptides reported that these peptides mainly act by activating PI3K/Akt/mTOR and MAPK signaling pathways while inhibiting protein degradation pathways, providing a strong rationale for the use of plant-derived peptides in sarcopenia intervention [21].

Given the multifactorial nature of sarcopenia, multimodal strategies that combine different nutritional interventions may offer greater therapeutic benefits than single-component approaches. Emerging evidence suggests that leucine supplementation, when combined with other nutrients, physical activity, or gut microbiota modulation, can enhance sarcopenia prevention and treatment beyond the effects of leucine alone [26]. Furthermore, the gut–muscle axis has gained increasing attention as a key modulator of muscle health, integrating the effects of nutrition, microbiota, and inflammation in the aging process [27]. Indeed, growing evidence underscores the need for multimodal interventions—combining optimized nutrition, exercise, and microbiota modulation—to maximize therapeutic benefits against sarcopenia [28].

Despite the promising individual effects of BDP and leucine, their mechanisms of action are clearly complementary. BDP primarily act by mitigating oxidative damage to myofibers and inhibiting protein degradation pathways through antioxidant and anti-inflammatory effects, thereby exerting a “damage-reducing” protective effect. In contrast, leucine directly activates mTORC1 signaling and enhances muscle protein synthesis, representing a “synthesis-promoting” anabolic effect. Theoretically, combining the two may simultaneously target both the “anabolic” and “anti-catabolic” arms of muscle metabolism, producing stronger anti-sarcopenic effects. However, no study has systematically evaluated the combined effect of BDP and leucine on sarcopenia. The combination of nutritional strategies targeting complementary pathways may represent a more effective approach for the prevention and management of sarcopenia than single-component interventions.

Therefore, in this study, we employed a D-galactose-induced aging mouse model to investigate whether BDP and leucine, alone or in combination, can ameliorate sarcopenia. By comprehensively assessing muscle mass, strength, function, and underlying molecular mechanisms through transcriptomic sequencing and qPCR validation, this study aims to provide a novel nutritional intervention strategy for sarcopenia and to elucidate the enhanced mechanisms of combined BDP and leucine supplementation.

## 2. Material and Methods

### 2.1. Animals

Male C57BL/6 mice (aged six weeks, weighing 20 ± 1 g) were obtained from SPF Biotechnology Co., Ltd. (Beijing, China). Following acquisition, the animals underwent a one-week acclimatization period with unrestricted access to standard chow and water. The housing environment was strictly controlled with a 12 h light/dark cycle, a temperature of 22 ± 2 °C, relative humidity between 40 and 70%, illuminance of 15–20 Lux, and an atmospheric pressure of 45 Pa. Upon completion of the acclimatization phase, the mice were randomly assigned to five experimental groups (*n* = 6 per group, each mouse as one experimental unit; total 30 mice): a normal control group, a sarcopenia model group, a BDP treatment group, a leucine treatment group, and a combined BDP and leucine treatment group.

To establish the sarcopenia model, mice in all groups except the normal control received a daily intraperitoneal injection of D-galactose (500 mg/kg body weight; Cat. No. G0750, Sigma-Aldrich, Darmstadt, Germany). Concurrently, the control and model groups were administered normal saline via daily oral gavage. The treatment groups received daily oral gavages of either BDP (200 mg/kg/day), leucine (500 mg/kg/day), or a combination of both, at the same respective dosages [29,30,31,32]. The gavage volume was consistently maintained at 100 μL per mouse. The intervention period lasted 8 weeks, during which all animals had ad libitum access to food and water, and their body weights were recorded weekly.

At the conclusion of the 8-week intervention, body composition and behavioral assessments were conducted on the mice in an awake state. Following these evaluations, all animals were humanely anesthetized by intraperitoneal injection of ketamine (75 mg/kg) and thiazine (8 mg/kg) and then euthanized with cervical dislocation. Key skeletal muscles, specifically the gastrocnemius and tibialis anterior, were promptly dissected and weighed. The skeletal muscle index for each muscle was subsequently calculated as the muscle weight normalized to the final body weight. For further analysis, muscle tissue samples were divided; one portion was fixed in 4% formalin for subsequent histopathological examination, while the remaining tissue was immediately flash-frozen in liquid nitrogen and stored at −80 °C for future molecular analyses. All experimental procedures were conducted in strict accordance with the ethical guidelines for animal research and were formally approved by the Animal Experiment Ethics Committee of China Agricultural University (Approval No. AW22215202-5-02).

All methods were performed in accordance with the ARRIVE guidelines and the guidelines of the Animal Experiment Ethics Committee of China Agricultural University.

### 2.2. Analysis of Body Composition

After the 8-week intervention period, body composition of the mice was measured using a small animal magnetic resonance imaging analyzer (QMR23-060H-I, Niumag, Shanghai, China). Each mouse was first weighed, and the weight was entered into the system prior to placement into the scanner for measurement. Upon completion of the scan, body composition data, including lean mass, fat content, and free water content, were obtained for each mouse.

### 2.3. Grid Hanging Test

These mice were suspended on an inverted grid positioned 60 cm above a thick soft pad. Each mouse was placed in the center of the grid, which was then gently inverted so that the mouse hung head-down. The hanging duration was recorded from placement until the mouse fell off the grid. Each mouse was tested twice with an interval of more than 30 min between tests. Hanging times were recorded and scored according to established criteria. If a mouse fell off within 10 s, the test was immediately repeated to ensure accuracy. This test relies on the mouse’s innate fear of falling; therefore, testing was performed under naive conditions with a limited number of repetitions.

### 2.4. Grip Strength Test

Forelimb grip strength was assessed using an electronic grip strength meter (Beijing Zhongshidichuang Science and Technology Development Co., Ltd., Beijing, China). The elastic metal bar was fixed to the sensor, which was then activated and set to peak mode before being calibrated to zero. Each mouse was gently grasped by the mid-tail region and placed on the elastic metal bar, allowing the front and back paws to firmly grip the bar while the trunk was maintained horizontally parallel to the bar. The tail was pulled backward gently and steadily until the mouse could no longer maintain its grip and released the bar. The maximum grip force was automatically recorded by the device. Each mouse was subjected to three consecutive measurements, and the maximum value was used for statistical analysis.

### 2.5. Endurance Test

(1) Adaptation Phase

The treadmill’s (Beijing Zhongshidichuang Science and Technology Development Co., Ltd., Beijing, China) parameters during the adaptation phase are presented in Table 1. The electrical stimulation current of the treadmill was set to 0.5 mA. A gradual training protocol was employed to acclimatize the mice to treadmill exercise. The adaptation was performed once daily for 2–3 days, with each session lasting approximately 10 min.

(2) Test Phase

The treadmill’s parameters during the test phase are presented in Table 2. The electrical stimulation current remained consistent at 0.5 mA. Exhaustion was defined as the cessation of running for 10 s, with no further response to additional electrical and noise stimulation for another 10 s. The time to exhaustion was recorded for each mouse. The test was conducted once daily for 2–3 consecutive days.

### 2.6. Hematoxylin–Eosin (HE) Staining for GAS

To evaluate the effects of BDP and leucine, both individually and in combination, on muscle tissue improvement, this study conducted histological examinations using HE staining and an optical microscope. The gastrocnemius muscle (GAS) was fixed in 4% paraformaldehyde for more than 48 h, trimmed to an appropriate size, and dehydrated to prepare paraffin blocks. A paraffin slicer (RM2255, Leica, Wetzlar, Germany) was used to cut the tissue into consecutive 5 μm thick sections, which were then dried overnight in a 60 °C oven. The paraffin sections were stained using an HE staining kit (G1120, Solarbio, Beijing, China) and then observed under an optical microscope (CTR6, Leica, Wetzlar, Germany). ImageJ software (v1.8.0, National Institutes of Health, Bethesda, MD, USA) was used to calculate and analyze data such as muscle fiber width.

### 2.7. RNA Sequencing (RNA-Seq)

Total RNA was extracted from mouse skeletal muscle tissue using Trizol reagent (Cat. No. CW0580S, Cwbio Co., Ltd., Beijing, China). A total of 15 biologically independent samples were sequenced, including three replicates per group: control (CK1-3), model (MOD1-3), leucine (LEU1-3), BDP (BDP1-3), and combination (COM1-3). RNA concentration, RIN value, 28S/18S ratio and fragment integrity were detected using an Agilent 2100 Bioanalyzer (Agilent Technologies, Santa Clara, CA, USA); all samples had RIN ≥ 7.0 and 28S/18S ratio ≥ 1.8. mRNA was enriched using Oligo (dT) magnetic beads (Invitrogen, Thermo Fisher Scientific, Waltham, MA, USA) and fragmented under high temperature. Using the fragmented mRNA as a template, first-strand and second-strand cDNA were sequentially synthesized. End repair, A-tailing and ligation of sequencing adapters were performed, followed by PCR amplification to construct the transcriptome library. After quality inspection, the library was subjected to high-throughput sequencing on the Illumina HiSeq platform (Illumina Inc., San Diego, CA, USA) (paired-end 150 bp).

Raw sequencing data were processed with SOAPnuke software (v1.5.2) to remove reads containing adapters, reads with >10% unknown N bases, and low-quality reads (defined as reads in which >20% of bases had a quality value < 15). After filtering, an average of 45.6 million clean reads per sample were obtained, with an average clean read ratio of 93.8%. The average Q20 and Q30 values were 99.5% and 96.8%, respectively (detailed per-sample statistics are provided in Table S1). Clean reads were mapped to the mouse reference genome using HISAT software (v0.1.6); the average mapping rate was 96.4%. Clean reads were aligned to the reference sequence using Bowtie2 software (v2.2.5), and gene and transcript expression levels were calculated using RSEM software (v1.2.12), with FPKM values as the quantitative indicator of gene expression. For downstream analyses, FPKM values were log2-transformed after adding a pseudocount of 1. Low-expression genes (mean FPKM < 1 across all samples) were filtered out before differential expression analysis.

Differentially expressed genes (DEGs) were screened using the PoissonDis algorithm with the criteria of false discovery rate (FDR) ≤ 0.001 and fold change ≥ 2 [33]. Principal component analysis (PCA) was performed using the prcomp function in R (v4.2.0) on the log2(FPKM+1) expression matrix to assess overall sample clustering. Gene Ontology (GO) functional enrichment analysis and Kyoto Encyclopedia of Genes and Genomes (KEGG) pathway enrichment analysis were performed on the identified DEGs. Enrichment calculation was performed using the phyper function in R software (R Foundation for Statistical Computing, Vienna, Austria), with FDR ≤ 0.1 as the threshold for significant enrichment. The analytical results were visualized using ggplot2 software (v3.4.3).

The PCA plot is provided in Figure S1. The complete list of DEGs for each comparison (|fold change| ≥ 2, FDR ≤ 0.001) is available in Table S2. Raw RNA-seq data have been deposited in the Gene Expression Omnibus (GEO); the accession number will be provided upon acceptance.

### 2.8. Quantitative Real-Time PCR and Western Blot

In this study, total RNA from mouse muscles was extracted from frozen samples using the Trizol reagent. Reverse transcription was carried out using the HiScript III RT SuperMix (Cat. No. R323-01, Vazyme Biotech Co., Ltd., Nanjing, Jiangsu, China) kit from Nanjing Novalza Biotechnology to synthesize cDNA. Subsequently, real-time fluorescence quantitative PCR was performed using the aq Pro Universal SYBR qPCR Master Mix (Cat. No. Q712-02, Vazyme Biotech Co., Ltd., Nanjing, Jiangsu, China) kit from Nanjing Noventa Biotechnology. The detection was carried out using the Applied Biosystems StepOnePlus real-time fluorescence quantitative PCR instrument (Model 7500, Applied Biosystems, Thermo Fisher Scientific, Waltham, MA, USA) from Thermo Fisher Scientific (Waltham, MA, USA). The mRNA expression changes in the target gene were calculated by the 2^−ΔΔCt^ method. In this experiment, actin (*Actb*) was used as the internal reference gene, and the expression levels of myogenic differentiation factor 1 (*Myod1*), myogenin (*Myog*), myocyte enhancer factor 2C (*Mef2c*), tumor necrosis factor-α (*Tnf*), myostatin (*Mstn*), C-X-C motif chemokine ligand 10 (*Cxcl10*), intercellular adhesion molecule 1 (*Icam-1*), sirtuin 3 (*Sirt3*) and apelin receptor (*Aplnr*) were analyzed. The primers used in the experiment were synthesized by Shenzhen BGI Genomics Co., Ltd. (please refer to Table S3).

For Western blot analysis, frozen skeletal muscle samples were collected and lysed in RIPA buffer containing PMSF (Beyotime Biotechnology, Shanghai, China) (RIPA:PMSF = 4:1, 200–400 μL). The tissue was homogenized on ice for 30 min, then centrifuged at 12,000 rpm for 15 min at 4 °C. The supernatant was collected, and the protein concentration was determined using a BCA assay kit (Beyotime Biotechnology, Shanghai, China).

Protein samples were mixed with loading buffer (at a 4:1 ratio and heated at 100 °C for 10 min. Following separation by SDS-PAGE (8–10%), proteins were transferred onto nitrocellulose membranes.

Membranes were blocked with 5% non-fat milk in TBST and then incubated overnight at 4 °C with primary antibodies at the indicated dilutions. After three washes with TBST, the membranes were incubated for 1 h with HRP-conjugated anti-rabbit IgG secondary antibody (Cat. No. 7074, Cell Signaling Technology (CST), Danvers, MA, USA).

Protein bands were visualized using enhanced chemiluminescence. The primary antibodies used were: anti-β-actin (66009-1-Ig, Proteintech, Rosemont, IL, USA),anti-MuRF1 (33973T, CST, Danvers, MA, USA), anti-Tnf-α (60291-1-Ig, Proteintech, Rosemont, IL, USA), anti-p-Erk (4370T, CST, Danvers, MA, USA), anti-Erk (4695T, CST, Danvers, MA, USA), anti-p-p70S6K (9205S, CST, Danvers, MA, USA), anti-p70S6K (9202S, CST, Danvers, MA, USA), and anti-mTOR (710216, Thermo Fisher, Waltham, MA, USA).

### 2.9. Preparation and Characterization of BDP

The BDP used in this study were prepared by enzymatic hydrolysis of broccoli seed protein. Briefly, broccoli seed protein was hydrolyzed using alcalase and flavourzyme, followed by ultrafiltration (3 kDa molecular weight cut off) to enrich the low molecular weight peptide fraction, and finally spray dried to obtain the final product. The batch used in this study (Lot No. 2025072010) is traceable. The Certificate of Analysis (COA) provided by the manufacturer showed that the peptide content was ≥99% (HPLC), protein content (dry basis) was 95.39%, and 98.85% of peptides had a molecular mass <1000 Da (HPLC size exclusion chromatography). The total nitrogen content was 15.87 g/100g (dry basis). Heavy metals (Pb, Cd, Hg, As) were below detection limits, and microbiological tests met food grade standards. (The key characteristics of the BDP used in this study are presented in Table S4.)

### 2.10. Statistical Analysis

Histological data were quantified using ImageJ software. All statistical analyses were performed with GraphPad Prism (v9.3.0, GraphPad Software, San Diego, CA, USA) and R software (v4.2.0). Before parametric testing, data normality was assessed using the Shapiro–Wilk test, and homogeneity of variances was evaluated using Levene’s test. All datasets met the assumptions of normality and equal variances; therefore, parametric tests were applied throughout.

For comparisons involving more than two groups, one-way analysis of variance (ANOVA) followed by Tukey’s post hoc test was used. Tukey’s post hoc test controlled the family-wise error rate at α = 0.05 for all pairwise comparisons. For the two-factor experimental design (BDP × leucine), two-way ANOVA was performed to evaluate main effects and interaction in the assessments of skeletal muscle indexes, exercise behavioral indicators, and muscle fiber cross-sectional area (CAS). A *p*-value < 0.05 was considered statistically significant. All data are presented as mean ± standard deviation (SD).

Sample size justification: Based on pilot experiment data, the required sample size was calculated using GPower software (v3.1.9.7, Heinrich-Heine-Universität Düsseldorf, Düsseldorf, Germany), and we therefore chose 6 mice per group.

## 3. Results

### 3.1. BDP and Leucine Increased Muscular Mass

**Figure 1:**
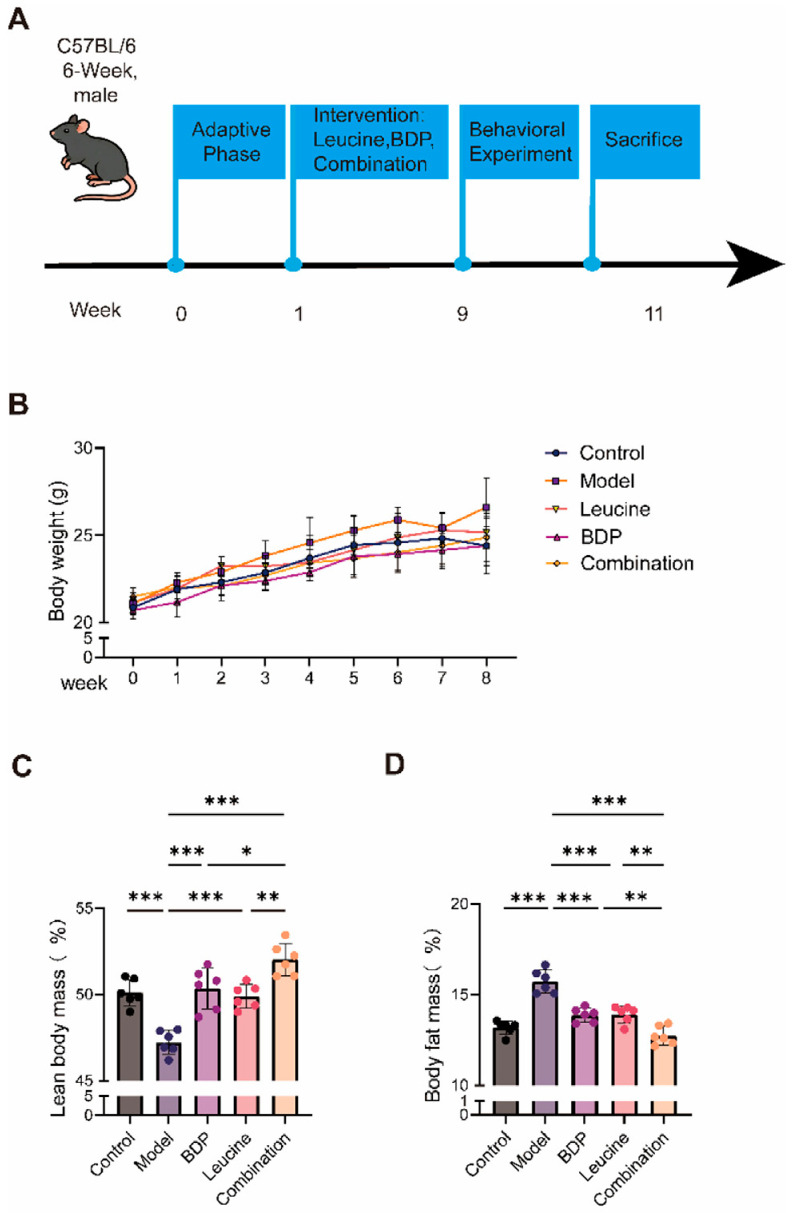
Improvement effect of BDP and leucine on body composition of mice with sarcopenia. (**A**) Experimental timeline. (**B**) Body weight of mice from the five groups. (**C**) Lean body content. Lean meat mass per mouse body weight ×100%. (**D**) Body fat content. Fat mass per mouse body weight. Results were presented as mean ± standard deviation. Each dot represents a mouse sample. Six animals per group. Statistically significant differences were determined by one-way ANOVA and Tukey’s post hoc test between groups (NS. *p* > 0.05, * *p* < 0.05, ** *p* < 0.01, *** *p* < 0.001).

In order to establish a mouse model of sarcopenia in this study, D-galactose was injected into C57BL/6 mice, and at the same time, an equal volume of normal saline, broccoli protein peptide, and leucine solution was administered intragastrically daily (Figure 1A). The weight changes in each group of mice during the intervention process showed a similar upward trend. Although mice in the model group exhibited a rising trend in body weight, the occurrence of sarcopenia was confirmed when integrated with subsequent muscle mass and functional data (Figure 1B). When body composition was examined in the awake state of mice, the results showed that the lean body mass of the model group was significantly reduced; the lean body mass of the BDP group, the leucine group, and the combination group all increased, among which the lean body mass of the BDP group and the combination group basically returned to the normal level or even exceeded the normal level (Figure 1B); the body fat mass of the BDP group, the leucine group, and the combination group was lower than that of the model group compared with it, and even the body fat mass of the combined group was lower than that of the control group (Figure 1C).

**Figure 2:**
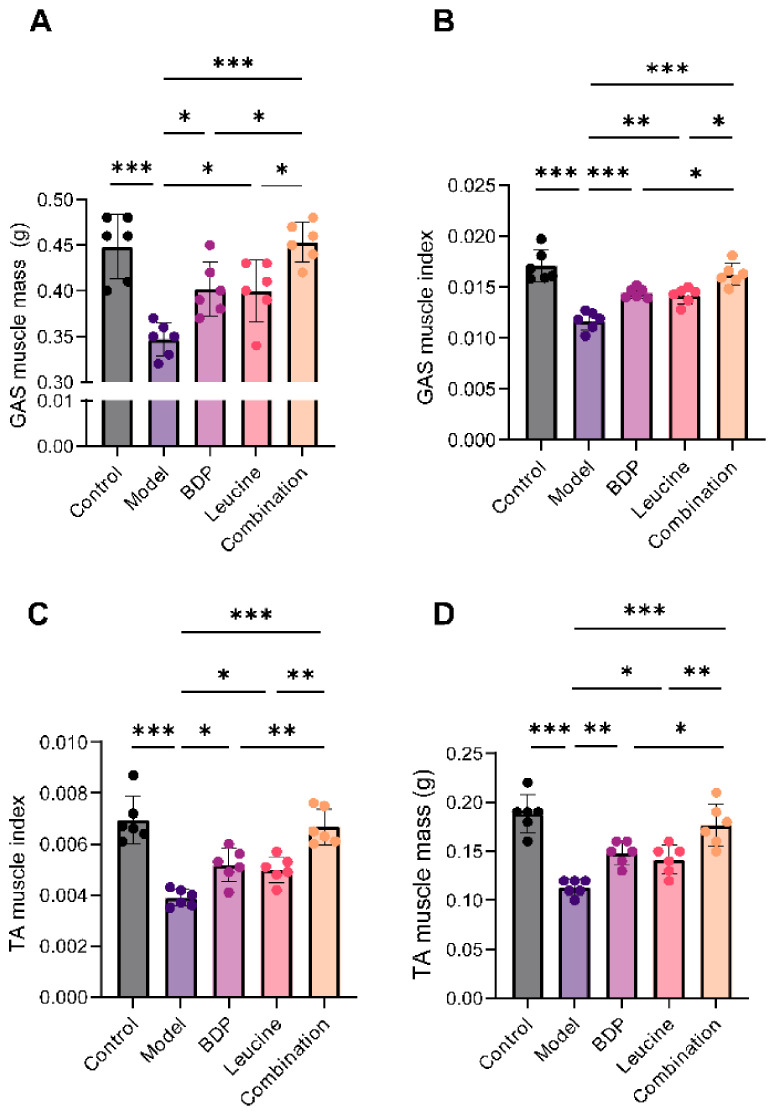
Effects of BDP and leucine on skeletal muscle of mice. (**A**) GAS muscle mass. (**B**) GAS muscle index. (**C**) TA muscle index. (**D**) TA muscle mass. Six animals per group. Statistically significant differences were determined by one-way ANOVA with Tukey’s post hoc test. Two-way ANOVA (BDP × leucine) showed no significant interaction for the endpoints shown (all *p* > 0.05). (NS. *p* > 0.05, * *p* < 0.05, ** *p* < 0.01, *** *p* < 0.001).

This study weighed the gastrocnemius muscle (GAS) and the tibialis anterior muscle (TA) of the skeletal muscles and calculated the corresponding skeletal muscle indices. The results showed that compared with the control group of mice, the muscle weight of the GAS and the TA in the model group of mice was significantly reduced (Figure 2A–D). The GAS and the TA muscle index are the criteria for judging muscle atrophy. The GAS muscle index in the hind limbs of the model group mice was significantly decreased, indicating that the D-galactose treatment successfully induced key sarcopenia-like phenotypes, which are consistent with previous reports using this accelerated aging model. Compared with the model group of mice, the mice in the BDP group, the leucine group, and the combination group had increased muscle weight and muscle index in the hind limbs, and the data of the combination group was higher than that of the BDP group and the leucine group. These indicate that the muscle quality of the mice improved after combined intervention. The above results show that BDP and leucine can alleviate the loss of skeletal muscle mass in mice, and the combined intervention effect is more significant.

### 3.2. BDP and Leucine Ameliorated Muscular Strength and Function

**Figure 3:**
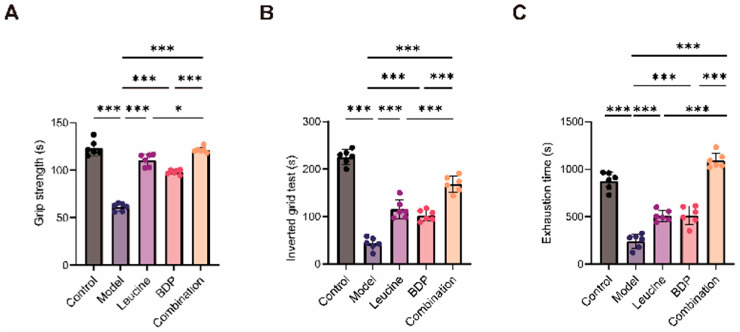
Each group’s performance on behavioral tests. (**A**) Forelimb grip strength assessment in mice. (**B**) The inverted hanging time of mice on a grid. (**C**) The exhaustion time of each group of mice in treadmill test. The results were presented as mean ± standard deviation. Six animals per group. Group comparisons were analyzed by one-way ANOVA with Tukey’s post hoc test. Two-way ANOVA was used to evaluate the interaction between Leu and BDP: significant interaction for A (*p* < 0.0001) and C (*p* = 0.0001), no significant interaction for B (*p* = 0.6410); main effects of both factors were significant (*p* < 0.0001) across all three indicators. (* *p* < 0.05, *** *p* < 0.001).

Sarcopenia is associated with progressive declines in skeletal muscle strength and functional capacity. To assess these parameters, we conducted standardized functional assessments in mice across experimental groups, including the strength of the grip (Grip strength test), inverted suspension time (Inverted grid test) and exhaustion time (Endurance test). Results revealed that the model group exhibited a statistically significant reduction in maximal grip strength relative to controls. In contrast, both leucine group and BDP group administration attenuated this decline, with the combination group yielding the most pronounced protective effect—maximal grip strength in the combination group was significantly higher than in either single-intervention group (Figure 3A). These findings suggest that combined leucine and BDP treatment confers superior enhancement of muscular strength compared with monotherapies.

Moreover, the inverted suspension duration and the exhaustion time served as complementary indicators of muscle endurance, demonstrating highly consistent trends across groups (Figure 3B,C). Specifically, the combination group showed a significantly prolonged inverted suspension duration and the exhaustion time relative to the model group—both differences reaching statistical significance. While either leucine or BDP alone improved endurance metrics relative to the model group, the combination intervention produced additive or stronger combination, indicating enhanced preservation and restoration of overall muscle function.

### 3.3. BDP and Leucine Improved the Condition of Muscle Tissue

**Figure 4:**
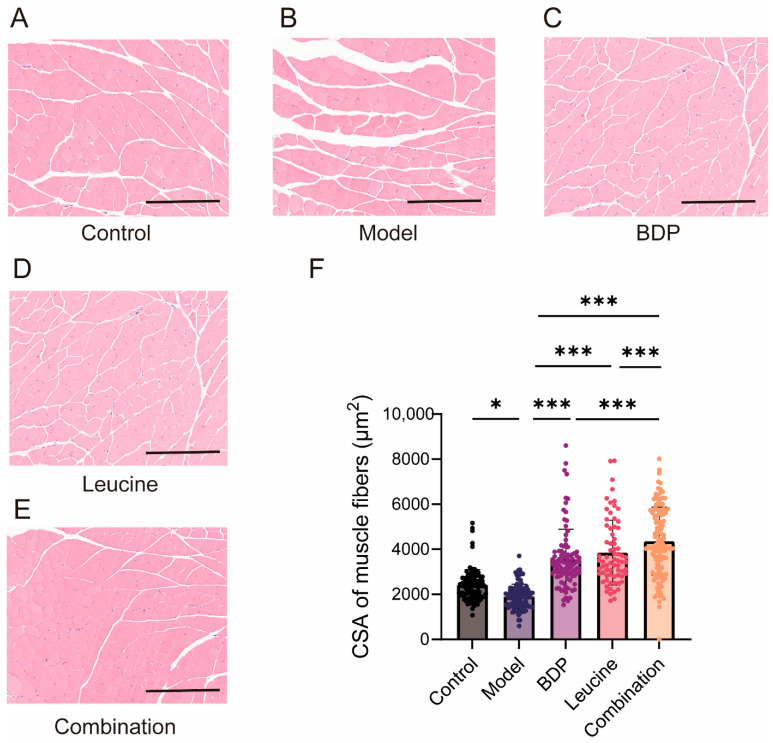
BDP and leucine improved the condition of muscle tissue. (**A**–**E**) HE staining of the gastrocnemius muscle of mice from Control (**A**), model (**B**), BDP (**C**), Leucine (**D**), and combination (**E**). Each image is representative of a typical GAS muscle. Scale bar: 200 µm. (**F**) CSA of muscle fibers. Statistically significant differences were determined by one-way ANOVA and Tukey’s post hoc test between groups. Two-way ANOVA was used to evaluate the interaction between BDP and leucine: a significant interaction was observed (*p* < 0.0001); main effects of both factors were significant (*p* < 0.0001). (* *p* < 0.05, *** *p* < 0.001).

The study evaluated the condition of the muscle tissue by conducting pathological staining on the skeletal muscle tissues of mice. From the hematoxylin-eosin staining (H&E), results of each group of mice, they can be seen that in the control group, the skeletal muscle fibers are closely arranged, with uniform diameters, and the cross-section of the fibers is polygonal, with the cell nuclei located at the edge of the cells. In the model group, the structure of the skeletal muscle fibers becomes locally loose and disordered, and the distance between the fibers increases. Compared with the model group’s mice, the cross-sectional area of the muscle fibers in the combination group increases, the cell arrangement is regular, the interstitial components are reduced, and the effects are significantly better than those of the BDP group and the leucine group (Figure 4A–E). This indicates that the combined intervention of BDP and leucine has a more obvious improvement effect on muscle atrophy than the individual interventions.

The study also further calculated the cross-sectional area (CSA) of the muscle fibers. The results showed that the CSA of the cells in the model group was significantly reduced; after supplementation with BDP and leucine, the CSA of the muscle cells in these two groups significantly increased compared to the model group; and the CSA of the muscle cells in the combination group was higher than that of the two individual intervention groups, and significantly higher than that of the control group (Figure 4F).

### 3.4. RNA-Seq in Mice Muscle Tissue

To identify differentially expressed genes (DEGs) involved in the anti-sarcopenic effects, we performed RNA-seq analysis on mouse skeletal muscle tissue. The discovery and subsequent analysis of these DEGs are instrumental in uncovering the underlying mechanisms associated with sarcopenia. PCA (Figure S1) confirmed clear separation between groups, with no outlier samples.

#### 3.4.1. Gene Ontology (GO) Enrichment Analysis

**Figure 5:**
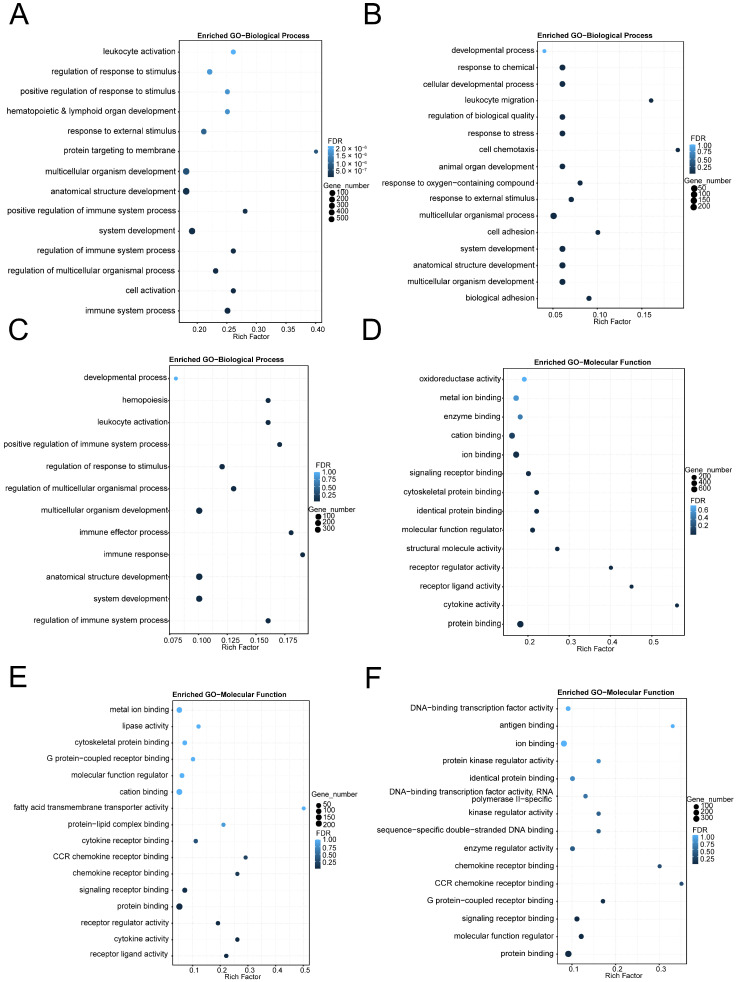
Transcriptome sequencing enriched sarcopenia-related GO biological process (BP) and molecular function (MF). Enriched GO BP of DEGs. The model was compared with group (**A**) BDP, (**B**) leucine, (**C**) combination. Enriched GO MF of DEGs. The model was compared with group (**D**) BDP, (**E**) leucine, (**F**) combination. The X-axis represents Rich Factor, and the Y-axis represents BP and MF names. The size of the point represents the number of DEGs. FDR ≤ 1.

GO enrichment analysis was performed to identify biological processes and molecular functions associated with the intervention groups (Figure 5). Compared with the model group, DEGs in the Leucine group were significantly enriched in biological processes related to cell adhesion, immune system process, and response to stress, as well as developmental processes such as anatomical structure development and multicellular organism development. At the molecular function level, the Leucine group showed strong enrichment in ion binding, cytoskeletal protein binding, and protein binding (Figure 5B,E).

The BDP group exhibited significant enrichment in biological processes including leukocyte activation, regulation of immune system process, and cellular developmental process. The molecular functions of this group were dominated by oxidoreductase activity, cytokine activity, receptor ligand activity, and structural molecule activity (Figure 5A,D).

The combination group displayed a broader range of enriched biological processes, covering most of the terms observed in both single-intervention groups, and additionally showed specific enrichment in regulation of cell movement, actin cytoskeleton organization, cell proliferation, and animal organ development. At the molecular function level, the combination group uniquely enriched DNA-binding transcription factor activity, protein kinase regulator activity, and G protein-coupled receptor binding (Figure 5C,F).

These results indicate that BDP and leucine, especially in combination, regulate multiple aspects of skeletal muscle cell development, immune function, cytoskeletal dynamics, and transcriptional control.

#### 3.4.2. KEGG Pathway Enrichment Analysis

**Figure 6:**
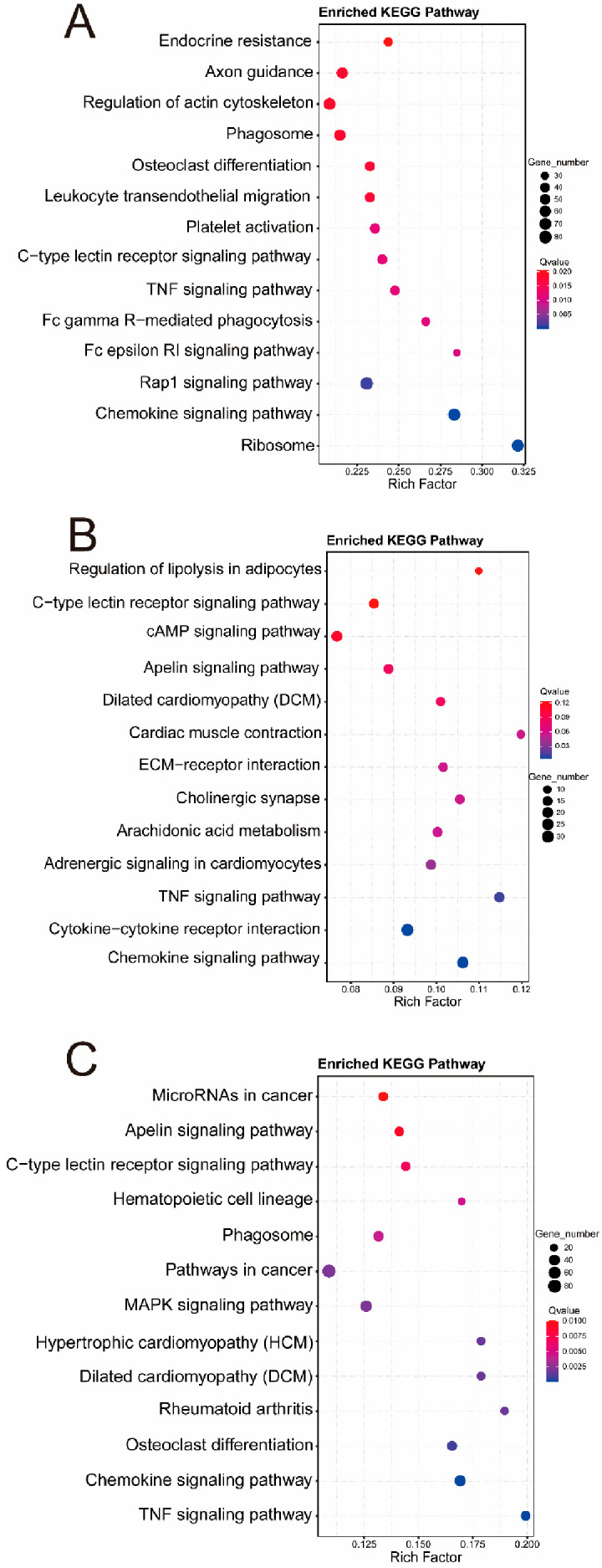
KEGG pathway enrichment analysis of BDP and leucine in sarcopenia. The model was compared with group (**A**) BDP, (**B**) leucine, (**C**) combination. The X-axis represents rich factor, and the Y-axis represents pathway names. The colors represent Q values. The size of the point represents the number of DEGs.

KEGG pathway enrichment analysis was performed to identify signaling pathways potentially involved in the anti-sarcopenic effects of BDP and leucine (Figure 6). The BDP group was significantly enriched in pathways including regulation of actin cytoskeleton, chemokine signaling, and TNF signaling (Figure 6A). The leucine group showed significant enrichment in Apelin signaling, ECM-receptor interaction, cardiac muscle contraction, and chemokine signaling (Figure 6B). Notably, the combination group uniquely enriched the MAPK signaling pathway, which was absent in either single-intervention group (Figure 6C). As reported in the literature, the MAPK signaling pathway is a unique regulator of cell proliferation, differentiation, and stress responses, and has been shown to play a critical role in skeletal muscle regeneration and metabolic adaptation [34,35]. Additionally, the TNF signaling pathway, while also present in the BDP group, was further enriched in the combination group, suggesting a superior effect on inflammatory regulation. These results suggest that the combined intervention may exert broader enhanced effects on muscle function by integrating inflammatory regulation, immune modulation, and metabolic signaling.

#### 3.4.3. Validation of the mRNA Expression Levels of Candidate Genes by qPCR

**Figure 7:**
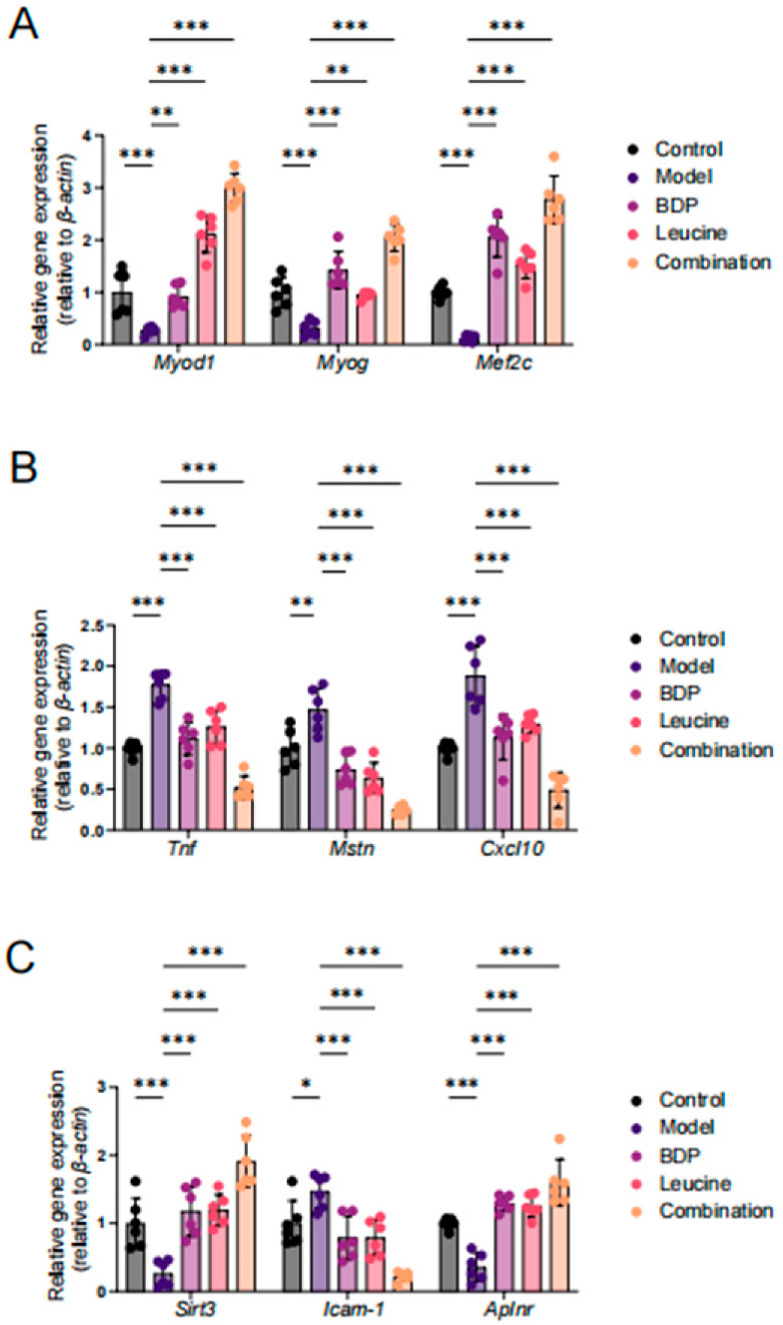
qPCR verified the expression changes in GO and KEGG pathway enriched sarcopenia-related DEGs and proteins. The mRNA expression of (**A**) *Myod1*, *Myog*, and *Mef2c*. (**B**) *Tnf*, *Mstn*, and *Cxcl10*. (**C**) *Sirt3*, *Icam1*, and *Aplnr*. relative to β-actin. Six animals per group. Statistically significant differences were determined by one-way ANOVA (* *p* < 0.05, ** *p* < 0.01, *** *p* < 0.001).

To validate the transcriptomic findings and quantitatively assess the effects of BDP and leucine on skeletal muscle, we examined the mRNA expression levels of nine candidate genes involved in myogenesis, inflammatory signaling, muscle protein degradation, cell adhesion, oxidative stress response, and energy metabolism by qPCR (Figure 7A–C).

*Myod1*, *Myog*, and *Mef2c* are core transcriptional regulators that govern skeletal muscle development, differentiation, and regeneration, with MRFs (*Myod1* and *Myog*) and MEF2 factors acting in a collaborative network to orchestrate the myogenic program [36]. The qPCR data revealed that the mRNA levels of *Myod1*, *Myog*, and *Mef2c* were significantly reduced in the model group compared with the control group. Treatment with BDP or leucine alone markedly upregulated these transcripts, and the combination group exhibited the highest expression levels, which were significantly higher than those in either single-intervention group (Figure 7A). These results are fully consistent with the GO enrichment of “developmental process”, “cellular developmental process”, and “DNA-binding transcription factor activity” in the BDP and combination groups (Figure 5A,C,F), indicating that combined BDP and leucine intervention robustly promotes the myogenic program in sarcopenic muscle.

*Tnf* (tumor necrosis factor-α) is a master pro-inflammatory cytokine that drives chronic low-grade inflammation (“inflammaging”) and contributes to muscle catabolism in sarcopenia [37]. A comprehensive review by Livshits and Kalinkovich highlighted that increased secretion of Tnf-α with aging may have a detrimental impact on muscle strength and mass during the progression of sarcopenia [8]. The qPCR data showed that *Tnf*’s expression was markedly elevated in the model group compared with the control group (Figure 7B). Treatment with BDP or leucine alone partially suppressed *Tnf*’s expression, whereas the combined intervention resulted in the most profound downregulation, aligning with the KEGG enrichment of the TNF signaling pathway in both the BDP and combination groups (Figure 6A,C).

*Mstn* is a well-characterized member of the transforming growth *factor-β* (TGF-β) superfamily, serves as a key negative regulator of skeletal muscle mass, and its overactivation is closely associated with the pathogenesis of various musculoskeletal and metabolic disorders [38]. The model group showed a significant increase in *Mstn*’s mRNA levels compared with the control group. Both BDP and leucine alone reduced Mstn expression, and the combination group exhibited the most pronounced suppression (Figure 7B), supporting the GO term “response to stress” (Figure 5A) and the anti-catabolic effect of the combined intervention.

*Cxcl10* (C-X-C motif chemokine ligand 10) is a key chemokine that mediates T-cell recruitment and has been reported to be selectively upregulated in aged skeletal muscle under stress conditions [39]. The KEGG analysis showed significant enrichment of the chemokine signaling pathway in all intervention groups (Figure 6A–C). Our qPCR validation confirmed that *Cxcl10*’s expression was significantly increased in the model group and profoundly suppressed by the combined intervention, while single interventions showed moderate but significant reductions (Figure 7B). These results indicate that BDP and leucine, especially in combination, effectively attenuate chemokine-driven inflammatory signaling in aged skeletal muscle.

*Sirt3* is a principal NAD^+^-dependent deacetylase localized primarily in mitochondria, exerts multimodal regulatory effects spanning mitochondrial bioenergetics, oxidative stress, and epigenetic modifications associated with aging [40]. A recent high-impact review by You and Wang comprehensively summarized that SIRT3 maintains mitochondrial redox homeostasis and counteracts oxidative stress-induced cell damage, playing a crucial role in aging-related diseases including sarcopenia [40]. The GO enrichment analysis revealed that the leucine group was significantly enriched for the molecular function term “oxidoreductase activity” (Figure 5E). Our qPCR results showed that *Sirt3*’s expression was significantly reduced in the model group compared with the control group. Both leucine alone and the combined intervention significantly upregulated *Sirt3*’s expression, with the combination group achieving the most pronounced restoration (Figure 7C). These results indicate that the combined intervention enhances mitochondrial antioxidant capacity.

*Aplnr* encodes the apelin receptor (APJ), is a key component of the apelin/APJ signaling system. A landmark study by Vinel et al. demonstrated that mice deficient in either apelin or APLNR presented dramatic alterations in muscle function with increasing age, and restoring apelin signaling considerably enhanced muscle function by triggering mitochondriogenesis and anti-inflammatory pathways in myofibers as well as enhancing regenerative capacity [41]. The KEGG pathway analysis showed significant enrichment of the Apelin signaling pathway in both the leucine group (Figure 6B) and the combination group (Figure 6C). Our qPCR data demonstrated that *Aplnr*’s expression was significantly decreased in the model group and restored by BDP and leucine interventions, with the combination group exhibiting the most marked upregulation (Figure 7C). This corroborates the KEGG enrichment findings and suggests that activation of the apelin/APJ axis contributes to the metabolic benefits of the combined nutritional intervention.

*Icam1* (intercellular adhesion molecule-1) is involved in immune cell trafficking and myoblast fusion. It was specifically enriched in the GO terms “cell adhesion” and “immune system process” (Figure 5A). A previous study by Buckley et al. demonstrated that ICAM-1 enhances myonuclear transcription during injury-induced muscle regeneration, leading to myofiber hypertrophy, and skeletal muscle cell expression of ICAM-1 augments myogenesis [42]. However, under chronic oxidative stress and inflammatory conditions induced by D-galactose, sustained downregulation of *Icam1* may contribute to pathological immune cell infiltration and exacerbate muscle inflammation. Our qPCR data showed that *Icam1* expression was significantly higher in the model group than in the control group. Treatment with BDP or leucine alone moderately reduced *Icam1* expression, and the combination group exhibited the most marked suppression, bringing its level close to or even below that of the control group (Figure 7C). Collectively, the qPCR validation results are highly consistent with the RNA-seq and GO/KEGG enrichment analyses (Figure 5, Figure 6 and Figure 7), supporting the reliability of our transcriptomic data. The combined BDP and leucine intervention effectively promotes myogenesis (upregulation of *Myod1*, *Myog*, *Mef2c*), suppresses chronic inflammation (downregulation of *Tnf*, *Cxcl10*), inhibits protein degradation (downregulation of *Mstn*), restores immune-adhesive function (downregulation of *Icam1*), enhances mitochondrial redox balance (upregulation of *Sirt3*), and activates energy metabolism signaling (upregulation of *Aplnr*). Notably, the superior efficacy of the combined intervention across these multiple complementary pathways aligns with the unique enrichment of the MAPK signaling pathway (Figure 6C), providing robust molecular evidence for the superior anti-sarcopenic effect of BDP and leucine.

### 3.5. Western Blot Validation of Key Proteins

**Figure 8:**
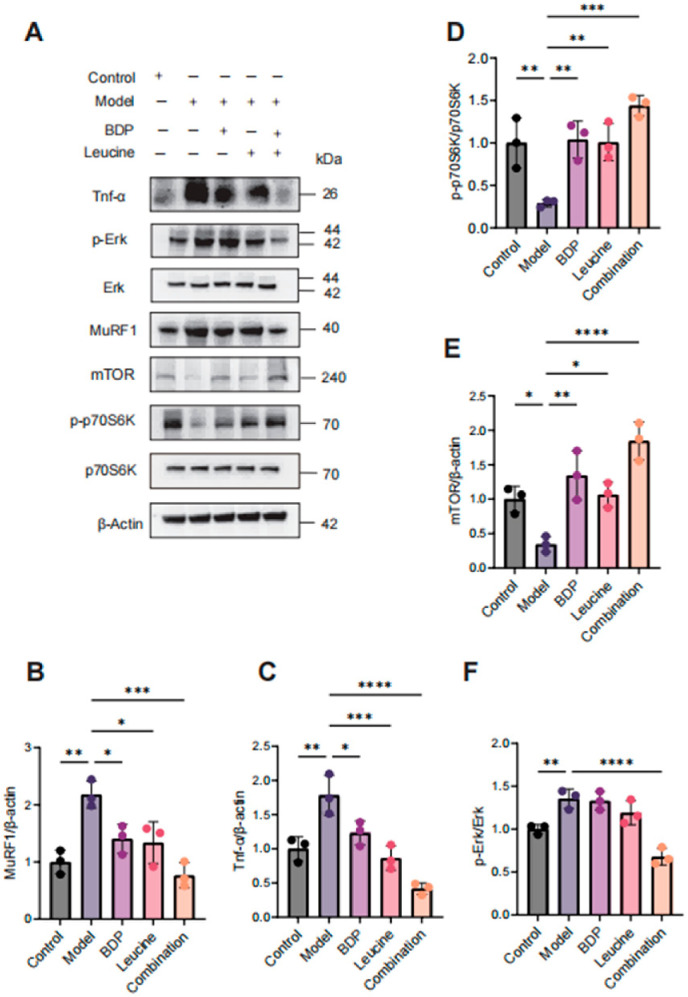
Western blot analysis of signaling proteins in mouse gastrocnemius muscle. (**A**) Representative blots of *p*-Erk, Erk, *p*-p70S6K, p70S6K, mTOR, MuRF1, Tnf-α, and β-actin. (**B**) Quantification of MuRF1 normalized to β-actin. (**C**) Quantification of Tnf-α normalized to β-actin. (**D**) Quantification of *p*-p70S6K/p70S6K ratio. (**E**) Quantification of mTOR normalized to β-actin. (**F**) Quantification of *p*-Erk/Erk ratio. Data are mean ± SD (*n* = 3 per group). (* *p* < 0.05, ** *p* < 0.01, *** *p* < 0.001, **** *p* < 0.0001).

To further validate the transcriptomic findings at the protein level, we examined the expression of key proteins involved in MAPK signaling, mTORC1 activity, muscle atrophy, and inflammation by Western blot (Figure 8).

As shown in Figure 8A, the combination of BDP and leucine markedly reduced the phosphorylation level of Erk compared with the model group, while total Erk remained unchanged. Quantitative analysis revealed that the *p*-Erk/Erk ratio was significantly lower in the combination group than in the model group (*p* < 0.05; Figure 8F), indicating that the combination treatment suppressed the excessive MAPK pathway activation induced by D-galactose [43].

Similarly, the combination treatment significantly elevated the *p*-p70S6K/p70S6K ratio relative to the model group (*p* < 0.05; Figure 8D), suggesting enhanced mTORC1 activity and protein synthesis capacity [44]. The total mTOR protein level was also significantly increased in the combination group compared with the model group (*p* < 0.05; Figure 8E), indicating enhanced mTOR expression. The activation of the mTORC1 signaling pathway was supported by the increased *p*-p70S6K/p70S6K ratio (Figure 8D).

Regarding muscle atrophy, the protein level of MuRF1 was markedly reduced in the combination group compared with the model group (*p* < 0.05; Figure 8B), consistent with an anti-catabolic effect. MuRF1 is a well-validated marker of muscle atrophy [45].

For inflammation, the combination treatment significantly suppressed Tnf-α expression relative to the model group (*p* < 0.05; Figure 8C), confirming reduced inflammatory status.

Together, these protein-level data corroborate the RNA-seq and qPCR results, supporting that the combined BDP and leucine intervention promotes anabolic signaling (mTORC1) while inhibiting excessive MAPK activation, catabolic, and inflammatory pathways.

## 4. Discussion

Sarcopenia is a multifactorial geriatric syndrome characterized by progressive loss of muscle mass, strength, and function, which severely impairs the quality of life in the elderly and imposes a heavy burden on healthcare systems [1,2]. In this study, we established a D-galactose-induced aging mouse model that mimics key features of sarcopenia, including reduced lean mass, decreased grip strength, impaired endurance, and diminished myofiber cross-sectional area, consistent with previous reports [21]. The successful model establishment allowed us to evaluate the anti-sarcopenic potential of broccoli-derived peptides (BDP) and leucine, alone or in combination.

Our primary finding is that both BDP and leucine supplementation significantly ameliorated muscle wasting and functional decline in sarcopenic mice, and notably, the combination of BDP and leucine produced superior effects across all parameters, including muscle mass, strength, and endurance. This superior effect aligns with the concept that multimodal nutritional strategies targeting complementary pathways may outperform single-component interventions [26,28].

Plant-derived bioactive peptides such as BDP possess anti-inflammatory and antioxidant properties [21,23], while leucine is well documented to promote protein synthesis via the mTORC1 pathway [14,15]. Although the mTOR pathway was not significantly enriched in the leucine group in our transcriptomic analysis, this is expected because mTOR signaling is primarily post-translationally regulated. Nevertheless, our Western blot analysis confirmed that the combination treatment significantly increased the *p*-p70S6K/p70S6K ratio (Figure 8D), supporting mTORC1 activation [44]. The total mTOR level was also elevated (Figure 8E), indicating increased mTOR protein expression.

Thus, leucine likely acts through multiple axes, including Apelin, and mTORC1 pathways. These findings suggest that the anti-sarcopenic mechanisms of leucine in the D-galactose-induced aging model may involve broader metabolic and regenerative signaling networks. Therefore, BDP is proposed to exert an “anti-catabolic” effect by mitigating oxidative damage and protein degradation, whereas leucine may act as a “pro-anabolic” regulator through multiple signaling axes. The combination simultaneously addresses both arms of muscle protein homeostasis, which likely underlies the observed additivity.

To dissect the molecular mechanisms, we performed transcriptomic analysis followed by GO and KEGG enrichment. GO analysis showed that BDP mainly influenced cell adhesion, immune system processes, and responses to stress, suggesting that its protective role involves maintaining the structural integrity of myofibers and modulating local inflammatory responses. These findings are in line with previous studies demonstrating that plant-derived peptides can alleviate muscle atrophy via anti-inflammatory and immunomodulatory mechanisms [24,25]. Recent reviews have also highlighted that harnessing immunomodulation represents a promising strategy to counteract sarcopenia, as age-related changes in immune function—including immune cell dynamics and chronic inflammation—are key drivers of disease progression [46]. In contrast, leucine-regulated GO terms were dominated by oxidoreductase activity and cytokine activity, highlighting its role in redox balance and intercellular signaling.

KEGG pathway analysis provided further mechanistic insight. The BDP group was enriched in regulation of actin cytoskeleton, chemokine signaling, and TNF signaling pathways, consistent with the hypothesis that BDP preserves cytoskeletal stability and attenuates chronic low-grade inflammation in aged muscle. The leucine group exhibited enrichment in Apelin signaling, ECM-receptor interaction, and cardiac muscle contraction. The Apelin signaling pathway is known to promote mitochondrial biogenesis and fatty acid oxidation in skeletal muscle [41], and the apelin/APJ system has been reported to activate PI3K/Akt to promote protein synthesis, suppress atrophy-related genes, and enhance regenerative capacity, while also modulating AMPK-mediated mitochondrial bioenergetics and NF-κB-driven inflammatory pathways [47]. ECM-receptor interaction is essential for maintaining muscle structural integrity [31]; dysregulation of the extracellular matrix can profoundly impair muscle mass and cross-sectional area, contributing to the onset of muscle atrophy [48]. These results support that leucine contributes to muscle health not only through protein synthesis but also through energy metabolism and extracellular matrix remodeling.

The combination group uniquely enriched the MAPK signaling pathway, which was absent in either the BDP or leucine group alone (Figure 6C). MAPK signaling is known to be a central regulator of cell proliferation, differentiation, and stress responses, and has been shown to play a critical role in skeletal muscle regeneration and metabolic adaptation [34]. A recent comprehensive review confirmed that exercise-regulated MAPK signaling networks are essential for mitochondrial biogenesis and the adaptive responses of skeletal muscle to metabolic challenges [35]. The unique enrichment of the MAPK pathway in the combination group suggests that BDP and leucine may contribute to stronger combined effects on muscle metabolism beyond their individual mechanisms. Additionally, the TNF signaling pathway, although also present in the BDP group, was further enriched in the combination group, suggesting an enhanced anti-inflammatory effect. The retention of Apelin signaling and C-type lectin receptor signaling in the combination group further is consistent with the multifaceted regulation of inflammation, immune modulation, and metabolic adaptation. However, these findings are correlational, and functional studies (e.g., using MAPK-specific inhibitors) are required to determine whether MAPK signaling is causally involved.

Our qPCR validation corroborated the transcriptomic results. The combination group significantly upregulated the myogenic regulatory factors *Myod1*, *Myog*, and *Mef2c*, which are core transcription factors orchestrating skeletal muscle development and regeneration [36]. Moreover, chronic inflammation markers *Tnf* and *Cxcl10* were effectively suppressed by the combined treatment, aligning with the KEGG enrichment of TNF and chemokine signaling pathways and supporting the concept that “inflammaging” is a key driver of sarcopenia [37]. The reduction in *Mstn* expression further indicated an anti-catabolic effect. Consistently, the protein level of MuRF1, a well-validated marker of muscle atrophy [45], was markedly reduced in the combination group (Figure 8B). Intriguingly, the combination group significantly suppressed *Icam1* overexpression induced by D-galactose. While ICAM-1 is beneficial for muscle regeneration under acute injury [42], its chronic upregulation in the context of sustained oxidative stress and inflammation may promote pathological immune cell infiltration and exacerbate muscle inflammation. Therefore, the marked downregulation of *Icam1* by the combined intervention likely reflects alleviation of chronic inflammatory stress rather than a loss of regenerative capacity. *Sirt3*’s upregulation in the leucine and combination groups correlates with the GO term “oxidoreductase activity” and reflects improved mitochondrial redox homeostasis [40], and a comprehensive sirtuin review confirms that SIRT3 plays a critical role in regulating skeletal muscle metabolism, including glucose uptake, fatty acid oxidation, mitochondrial dynamics, and autophagy regulation [49]. *Aplnr* upregulation in the combination group mirrors the KEGG enrichment of the Apelin signaling pathway and, together with a landmark study by Vinel et al. [41], supports the notion that restoring apelin/APJ signaling enhances muscle function through mitochondriogenesis and anti-inflammatory mechanisms.

Collectively, our results demonstrate that BDP and leucine, especially in combination, combat sarcopenia through a network of complementary pathways: promoting myogenesis (*Myod1*/*Myog*/*Mef2c*), reducing inflammation (*Tnf*/*Cxcl10*), inhibiting protein degradation (*Mstn*), attenuating excessive immune-adhesive signaling (downregulation of *Icam1*), improving mitochondrial redox balance (*Sirt3*), and activating energy metabolism (*Aplnr*). The unique enrichment of the MAPK pathway in the combination group suggests a potential distinction from single components, which warrants further investigation.

Compared with other combined nutritional interventions for sarcopenia, such as lactoferrin combined with creatine [31] or CoQ10, our study is the first to demonstrate a stronger anti-sarcopenic effect between a plant-derived peptide and an amino acid. While those studies highlighted pathways like longevity regulation, focal adhesion, and ECM-receptor interaction, they also reported enrichment of mTOR/PI3K-Akt and insulin resistance pathways in the combination groups–pathways that were not enriched in our KEGG analysis. This disparity may reflect the distinct properties of plant-derived nutrients versus animal-derived nutrients. Nonetheless, the consistent observation that a combined nutritional strategy yields broader pathway enrichment and superior efficacy across studies supports the general principle of multimodal intervention.

We acknowledge that the D-galactose model used in this study is an accelerated aging model, and as discussed in the Limitations section, our findings should be translated to naturally occurring sarcopenia with caution.

Nevertheless, our protein-level evidence (*p*-Erk, mTOR, *p*-p70S6K, MuRF1) directly supports the multimodal anti-sarcopenic effect of BDP and leucine, consistent with studies showing that targeting MAPK and mTOR signaling pathways holds promise for sarcopenia intervention [43,44].

In conclusion, this study demonstrates that BDP and leucine treatment is more effective than either agent alone in ameliorating D-galactose-induced sarcopenia through complementary mechanisms: BDP primarily exerts anti-catabolic effects by attenuating oxidative damage and inflammatory signaling, while leucine enhances energy metabolism and mitochondrial redox balance through Apelin and oxidoreductase-related pathways. The combined intervention uniquely enriched the MAPK signaling pathway in transcriptomic analysis, suggesting a potential involvement of this pathway in the enhanced anti-sarcopenic effect. Nevertheless, functional validation is required to establish causality. Furthermore, our Western blot validation (*p*-Erk, mTOR, *p*-p70S6K, MuRF1) directly supports the suppression of excessive MAPK signaling and activation of anabolic pathways by the combination treatment [43,44]. These findings establish BDP combined with leucine as a potential multimodal nutritional strategy targeting the multifactorial pathogenesis of sarcopenia, and provide a rationale for developing plant-based, multi-component interventions against age-related muscle decline.

## 5. Conclusions

BDP and leucine supplementation, alone or in combination, ameliorated D-galactose-triggered sarcopenic manifestations including decreased muscle mass, impaired physical performance and myofiber atrophy, with combined intervention showing the most prominent benefits. Transcriptomic analysis indicated differential pathway regulation by two ingredients, and only co-administration uniquely regulated MAPK signaling. qPCR and western blot results demonstrated that their combination simultaneously promoted myogenic differentiation, improved mitochondrial energy metabolism, and inhibited muscle inflammation and protein degradation via synergistic anti-catabolic and anabolic mechanisms. This multimodal nutritional combination provides a promising plant-based strategy to counteract age-related muscle wasting, though further research using naturally aged animal models is needed to support clinical translation.

## 6. Limitation

The first limitation of this study is that the D-galactose-induced aging model represents an accelerated oxidative stress and aging model rather than a full recapitulation of naturally occurring geriatric sarcopenia. As acknowledged in the literature, D-galactose is widely used to induce accelerated aging phenotypes in rodents through oxidative stress and mitochondrial dysfunction, but it does not fully capture the multifactorial complexity of age-related sarcopenia in elderly individuals. Therefore, while our findings demonstrate that the combination of BDP and leucine effectively ameliorates muscle atrophy in this accelerated aging model, the translation of these results to naturally occurring sarcopenia should be made with caution. Future studies using naturally aged mice are warranted to validate the efficacy of this nutritional intervention.

For RNA-seq analysis, three biological replicates per group were used, which is the minimal standard. However, all key DEGs were validated by qPCR using six biological replicates per group (Figure 7), and PCA (Figure S1) showed good group separation, supporting data robustness.

Due to technical limitations, amino acid composition and peptide profiling of BDP were not performed in this study. Further characterization of this component will be conducted in future studies.

## Figures and Tables

**Table 1:** Parameters of treadmill in the adaptation stage.

	Speed (m/min)	Acceleration Time (s)	Velocity Duration (min)
Initial velocity	5	5	1
First-order velocity	10	5	4
Second-order velocity	15	5	2

**Table 2:** Parameters of treadmill in the test stage.

	Speed (m/min)	Acceleration Time (s)	Velocity Duration (min)
Initial velocity	5	5	1
First-order velocity	15	5	exhaustion

## Data Availability

The raw data supporting the conclusions of this article will be made available by the authors on request.

## Supplementary Materials

The following supporting information can be downloaded at: https://www.mdpi.com/article/10.3390/nu18121997/s1, Figure S1: PCA plot showing the clustering of samples from different groups; Table S1: RNA-seq quality metrics per sample; Table S2: Complete list of differentially expressed genes; Table S3: Primers used for quantitative real-time PCR; Table S4: Characterization of broccoli-derived peptides (BDP).

## Abbreviations

BDP	Broccoli-derived peptides
CSA	Cross-sectional area
DEGs	Differentially expressed genes
ECM	Extracellular matrix
FDR	False discovery rate
GAS	Gastrocnemius
GO	Gene Ontology
HE	Hematoxylin-eosin
KEGG	Kyoto Encyclopedia of Genes and Genomes
MAPK	Mitogen-activated protein kinase
MRFs	Myogenic regulatory factors
mTOR	Mammalian target of rapamycin
qPCR	Quantitative real-time PCR
RNA-seq	RNA sequencing
ROS	Reactive oxygen species
TA	Tibialis anterior
TNF	Tumor necrosis factor
