# Supplementary material for: Broccoli-Derived Peptides and Leucine in Combination Ameliorate D-Galactose-Induced Sarcopenia in Mice
Source: Nutrients. 2026 Jun 19;18(12):1997. doi: 10.3390/nu18121997 (PMC13306220; doi:10.3390/nu18121997)
Supplement: Supplementary file 1 [file nutrients-18-01997-s001.zip › Table S3.pdf]

Table S3. Primers designed for quantitative Real-Time PCR .

Primer	Sequence(5' to 3')
β-actin Forward	TGAGCTGCGTTTTACACCCT
β-actin Reverse	GCCTTCACCGTTCCAGTTTT
Myod1 Forward	AACTGTCCTTTCGAAGCCGT
Myod1 Reverse	TTGGGGCTGGATCTAGGACA
Myog Forward	CAGCCCAGCGAGGGAATTTA
Myog Reverse	AGAAGCTCCTGAGTTTGCCC
Mef2c Forward	GCACCAACAAGCTGTTCCAG
Mef2c Reverse	CTGAATCGTCTGCATCGGGA
Tnf Forward	CTGGATGTCAATCAACAATGGGA
Tnf Reverse	ACTAGGGTGTGAGTGTTTTCTGT
Mstn Forward	AGTGGATCTAAATGAGGGCAGT
Mstn Reverse	GTTTCCAGGCGCAGCTTAC
Cxcl10 Forward	CCAAGTGCTGCCGTCATTTTC
Cxcl10 Reverse	GGCTCGCAGGGATGATTTCAA
Sirt3 Forward	GTCCGGGAGTGTTACAGGTG
Sirt3 Reverse	ACCATGACCACCACCCTACT
Icam1 Forward	TTCTCATGCCGCACAGAACT
Icam1 Reverse	TCCTGGCCTCGGAGACATTA
Aplnr Forward	CAGACGCCTCGGAAAATGG
Aplnr Reverse	CAGCGATGGTTTGGGCAATG
