# Supplementary material for: Broccoli-Derived Peptides and Leucine in Combination Ameliorate D-Galactose-Induced Sarcopenia in Mice
Source: Nutrients. 2026 Jun 19;18(12):1997. doi: 10.3390/nu18121997 (PMC13306220; doi:10.3390/nu18121997)
Supplement: Supplementary file 1 [file nutrients-18-01997-s001.zip › Table S4.pdf]

Supplementary Table S4. Characterization of broccoli-derived peptides (BDP) used in this study (Lot No. 2025072010).

Parameter	Result	Method
Appearance	Powder, loose, no agglomeration, no visible impurities	Visual inspection
Odor and taste	Pure and rich product aroma, no off-odor	Sensory evaluation (GB 7101)
Particle size	100% pass through 80-mesh sieve	Sieve analysis
Moisture (%)	3.76	GB 5009.3
Ash (%)	1.22	GB 5009.4
Protein content (%, dry basis)	95.39	GB 5009.5
Total nitrogen (g/100g, dry basis)	15.87	GB 5009.5
Peptide content (%, dry basis)	99.65	HPLC
Molecular weight distribution: <1000 Da (%)	98.85	HPLC size-exclusion chromatography
Number-average molecular weight (Mn, Da)	893	GPC
Weight-average molecular weight (Mw, Da)	2341	GPC
Polydispersity (Mw/Mn)	2.62	GPC
Lead (Pb, mg/kg)	<0.02	GB 5009.12

Parameter	Result	Method
Cadmium (Cd, mg/kg)	<0.02	GB 5009.15
Total mercury (Hg, mg/kg)	<0.02	GB 5009.17
Total arsenic (As, mg/kg)	<0.02	GB 5009.11
Total plate count (CFU/g)	<10	GB 4789.2
Escherichia coli (CFU/g)	<10	GB 4789.3
Yeast and mold (CFU/g)	<10	GB 4789.15
Pathogens (Salmonella, S. aureus)	Not detected	GB 4789.4 / GB 4789.10
