# Supplementary figures and images for: Broccoli-Derived Peptides and Leucine in Combination Ameliorate D-Galactose-Induced Sarcopenia in Mice
Source: Nutrients. 2026 Jun 19;18(12):1997. doi: 10.3390/nu18121997 (PMC13306220; doi:10.3390/nu18121997)

A

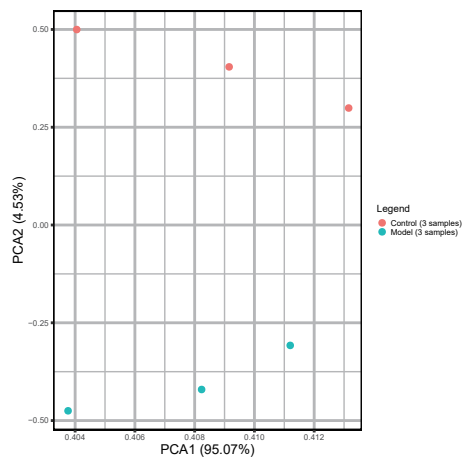

B

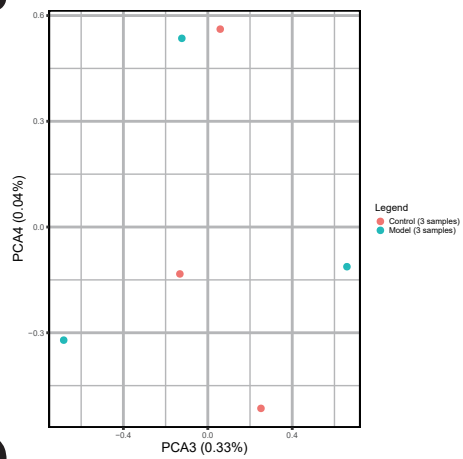

C

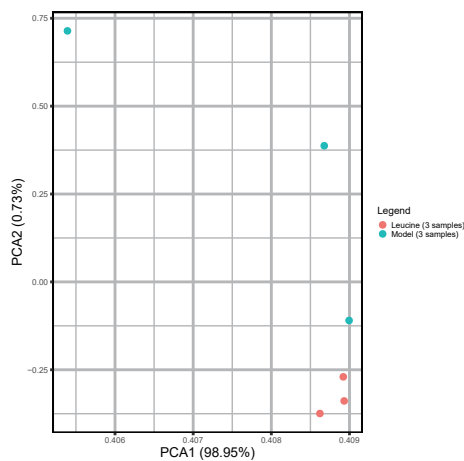

D

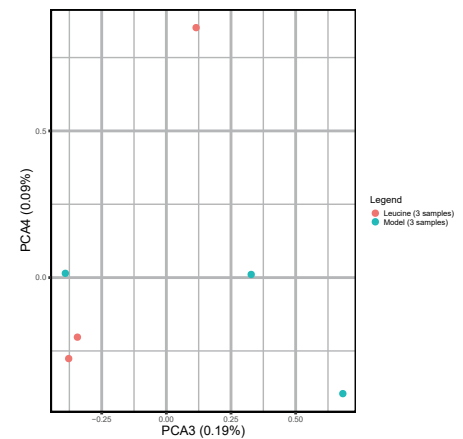

E

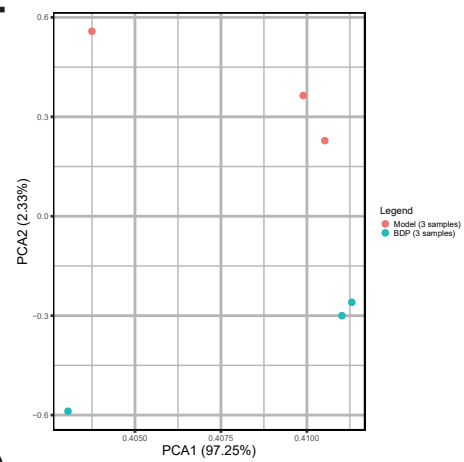

F

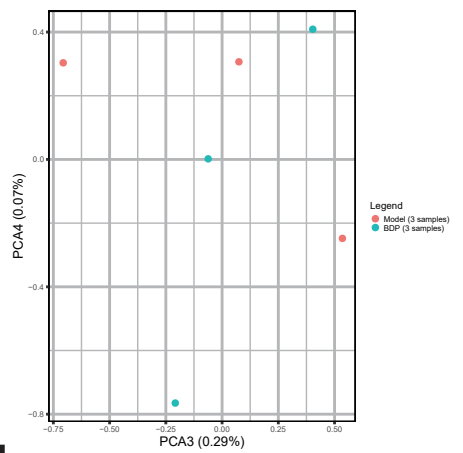

G

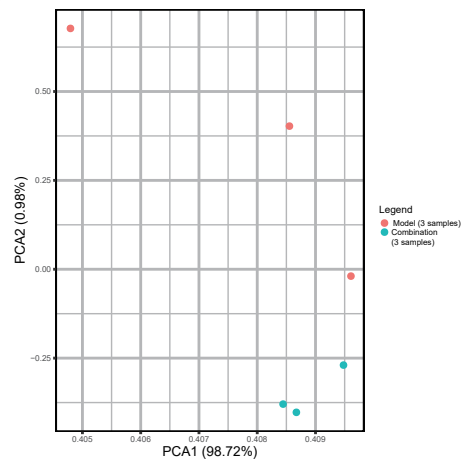

H

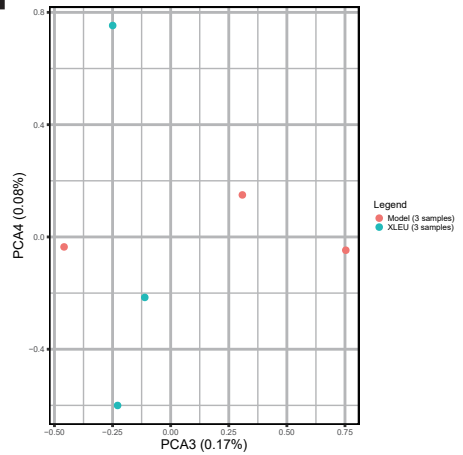

Supplement: Supplementary file 1 [file nutrients-18-01997-s001.zip › Figure S1.PCA plot showing the clustering of samples from different groups.pdf]
